# Optical Ultracompact Directional Antennas Based on a Dimer Nanorod Structure

**DOI:** 10.3390/nano12162841

**Published:** 2022-08-18

**Authors:** Fangjia Zhu, María Sanz-Paz, Antonio I. Fernández-Domínguez, Mauricio Pilo-Pais, Guillermo P. Acuna

**Affiliations:** 1 Department of Physics, University of Fribourg, Chemin du Musée 3, CH-1700 Fribourg, Switzerland; 2 Departamento de Física Teórica de la Materia Condensada and Condensed Matter Physics Center (IFIMAC), Universidad Autónoma de Madrid, E-28049 Madrid, Spain

**Keywords:** nanoantenna, plasmonics, ultracompact, metallic nanorod, unidirectionality

## Abstract

Controlling directionality of optical emitters is of utmost importance for their application in communication and biosensing devices. Metallic nanoantennas have been proven to affect both excitation and emission properties of nearby emitters, including the directionality of their emission. In this regard, optical directional nanoantennas based on a Yagi–Uda design have been demonstrated in the visible range. Despite this impressive proof of concept, their overall size (~λ^2^/4) and considerable number of elements represent obstacles for the exploitation of these antennas in nanophotonic applications and for their incorporation onto photonic chips. In order to address these challenges, we investigate an alternative design. In particular, we numerically study the performance of a recently demonstrated “ultracompact” optical antenna based on two parallel gold nanorods arranged as a side-to-side dimer. Our results confirm that the excitation of the antiphase mode of the antenna by a nanoemitter placed in its near-field can lead to directional emission. Furthermore, in order to verify the feasibility of this design and maximize the functionality, we study the effect on the directionality of several parameters, such as the shape of the nanorods, possible defects in the dimer assembly, and different positions and orientations of the nanoemitter. We conclude that this design is robust to structural variations, making it suitable for experimental upscaling.

## 1. Introduction

Microscopic light emitters, such as atoms, molecules, or quantum dots, hold great promise for quantum information applications [1] and photonic chips [2]. However, to achieve this, not only ultrabright [3] but also directional photon sources [4,5] are required. Optical nanoantennas act as effective transducers between the near- and far-field regions of these nanoemitters, and as such they have been widely applied for manipulating the interaction between light and matter [6]. To date, several schemes have been used to engineer the emitter properties, including tuning excitation [7], decay rate [8], polarization [9], frequency conversion [10,11], spectral modulation [12], nonlinear processes [13] and emission direction [14,15]. The most commonly used design for directional emission is based on the Yagi–Uda geometry [14,15,16,17] inspired by radiofrequency devices. There are also other designs proposed to achieve directional emission or scattering in the visible range, ranging from a pair of bimetallic nanodisks [18,19], or V-antennas [20,21], to trimers [22] and a nanorod standing on a disk [23]. However, the large size of these metal antennas may introduce high absorption losses, and the accompanying Joule heating causes dysfunction of nearby temperature-dependent devices [24]. For example, the Yagi–Uda antenna is based on the far-field interference between the electromagnetic waves produced by a feed, a reflector and several directors, spanning an area in the order of λ^2^/4, due to the number of elements and the specific gaps between them [25]. Furthermore, these geometric constraints, including the precise emitter positioning, requires demanding and serial top-down fabrication techniques, such as electron or ion beam lithography [16,26], which are not accessible to common chemical laboratories.

All these shortcomings call for an alternative compact antenna design allowing the integration of directional nanoemitters into photonic chips. Pakizeh and Käll have theoretically proposed an ultracompact antenna design that is based on a stacked gold nanodisk dimer [27]. In this case, directionality is achieved by exciting the antiphase plasmon mode through a localized emitter [28]. Similarly, Shen et al. used nanostrip dimers embedded on a dielectric material to induce directionality [29] and achieved a compact plasmonic-diamond hybrid nanostructure [30]. On the other hand, Bonod et al. have proposed a different design, composed by two coupled nanospheres [31]. Both structures achieve directionality by adjusting phase differences introduced by mode hybridization and optical path difference, respectively. At the same time, other theoretical proposals, based on dielectric or hybrid nanostructures [32,33,34], phase-change materials [35] or plasmonic structures supporting magnetic modes [36], are predicted to achieve directional emission by using the far-field superposition of electric and magnetic dipoles implementing the Kerker condition [37]. Still, to date experimental studies on compact directional optical antennas addressing single emitters are limited.

Recently, we have realized experimentally, for the first time, the directional ultracompact antenna design originally proposed by Pakizeh and Käll [38]. Here, we present a numerical study of the performance of these antennas using Finite Element Method (FEM) simulations on gold nanorods (AuNRs) of sizes and shapes in accordance with our experimental samples. In order to analyze the robustness and versatility of this design, and maximize the directionality, we take several factors into consideration, e.g., common fabrication limitations like nanoparticles with commercially available dimensions, simplicity (reducing the number of needed elements), coupling to emitters and optimization of footprint. We also considered the effect of geometrical parameters such as the gap size between AuNRs or the position and orientation of the nanoemitter. By converting far-field signals to back focal plane (BFP) images, we quantified the directionality of the antennas with a forward to backward power ratio (*F*/*B*). Finally, we used an analytical two-dipole model to explain the mechanism behind the numerical results and quantified the phase difference between the fields at both AuNRs. Overall, our numerical results indicate that the ultracompact antennas show excellent and robust directionality, with a *F*/*B* value that can be as large as 14.2 dB.

## 2. Materials and Methods

A frequency domain solver based on FEM in CST Studio Suite was used for the 3D full-wave simulation.

For the model without a substrate, the boundaries were set to open (add space) in the six faces. In the presence of a substrate, the boundaries were also open except for the plane wave input surface for simulating semi-infinite substrate. The refractive index (*n*) of air, water and glass were set to *n* = 1, 1.33 and 1.5, respectively. The dielectric function of gold corresponded to the fitting data from Johnson and Christy [39].

In the far-field simulations with substrate, the size of the glass was 1000 × 1000 × 500 nm (length × width × thickness). A discrete port with 5000 ohms combined with a Hertzian dipole (point dipole) was simulated as a single nanoemitter.

For calculating the scattering spectra of AuNR with a glass substrate, the size of the substrate was reduced to 400 × 400 × 150 nm. The whole model was used to calculate the total electric field and the magnetic field, and the model without AuNRs was used to calculate the background electric and magnetic fields. The final scattering fields obtained by subtracting the background field from the total field were used to obtain the scattered power and cross–section. The scattering spectra were averaged from the results obtained under excitation by two plane waves with orthogonal polarization at normal incidence.

For calculating the orientation average of the nanoemitter and the sum of radiated power at different wavelengths, the radiated power of the antennas was first normalized by their accepted power (sum of radiated power and nonradiated power) and then the arithmetic mean was taken.

According to Gauss’ law, the surface charge density can be obtained by *ρ* = ε_0_(**n**∙**E**) = ε_0_(n_x_E_x_ + n_y_E_y_ + n_z_E_z_) [40]. Then, the dipole moments distribution of AuNRs along the x axis can be calculated by $p_{i}(x,\;\lambda )=\iint_{NR_{i}(x,\;\lambda )}\rho (x,\;y,\;z,\;\lambda )y\;dydz$ and total dipole moments of AuNRs can be calculated by $P_{i}(\lambda )=\int_{NR_{i}(\lambda )}p_{i}(x,\;\lambda )\;dx$. Notice that dipole moments are complex values here [20]. Consequently, after discretization, we observe that $\sum_{x}\left|p_{i}\right|\neq \left|P_{i}\right|$.

## 3. Results

**Figure 1:**
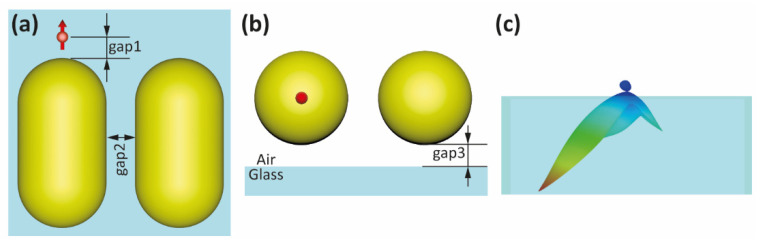
(**a**,**b**) Sketches of the ultracompact nanoantenna based on two AuNRs and a single dipolar nanoemitter (red arrow) on a glass substrate operating at a wavelength λ. (**c**) Corresponding radiation pattern when gap1, gap2 and gap3 are set to 5 nm and at λ = 570 nm. Color scale represents intensity.

The main parameters considered in the analysis of the ultracompact antenna performance are depicted in Figure 1. Two parallel AuNRs form a dimer in a side-to-side configuration. A nanoemitter, modelled as a point-dipole light source operating at a wavelength λ, is positioned above the tip of one of the AuNRs at a distance gap1, while the two AuNRs are separated by a distance gap2. These two AuNRs make up the ultracompact nanorod dimer antenna (NRDA) studied in this work, where directional emission can be obtained under near-field excitation by the nanoemitter. For comparison, we also study a single AuNR coupled to a nanoemitter placed at its tip (i.e., without the right AuNR in Figure 1a). This structure is hereafter referred to as a nanorod monomer antenna (NRMA). Both the NRDA and NRMA are placed on the top of a glass substrate, in agreement with typical experimental conditions. The distance from the AuNRs to the glass surface is gap3, as shown in Figure 1b.

For the initial FEM far-field simulations, we choose AuNRs with commercially available sizes and similar to the experiments in Ref. [38]: 40 nm diameter (2R), 68 nm length (L) and ideal semi-sphere caps. Distances gap1 and gap2 are set to 5 nm. The gap3 is also set to 5 nm to account for eventual functionalization of the substrate or the AuNRs. The background medium employed is vacuum (*n* = 1), however, an exemplary simulation including the effect of the ligands for self-assembly such as DNA are included in Figure S6. Unless specified otherwise, these are the parameters for all FEM simulations. The results show that the radiation pattern of the NRDA is asymmetric within a specific wavelength range around 570 nm (see Figure 1c), with the main emission lobe occurring at the side of the antenna where the emitter is placed. As will be discussed later, the wavelength range where directionality occurs corresponds to the antiphase plasmon mode of the NRDA.

**Figure 2:**
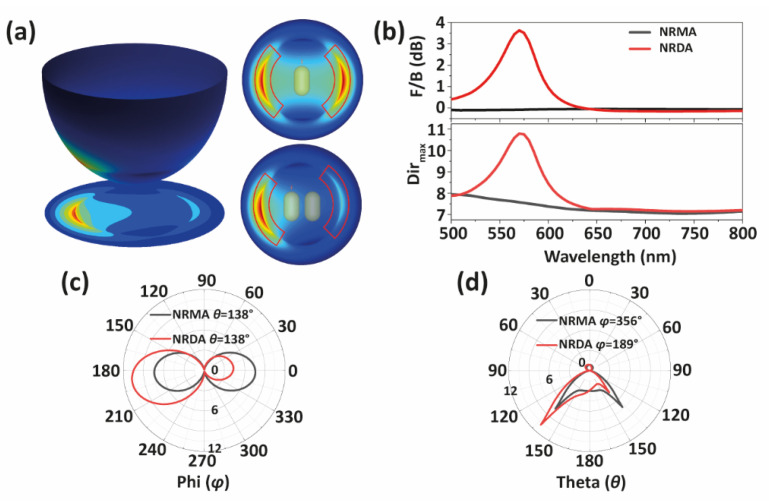
(**a**) NRDA radiation on a hemispherical surface and its projection on the back focal plane (**left**), back focal plane images of NRMA and NRDA at λ = 570 nm (wavelength of maximum directivity) (**right top** and **right bottom** insets respectively). (**b**) *F*/*B* ratio and *Dir_max_* of NRMA and NRDA on air–glass interface. (**c**,**d**) Azimuthal and polar radiation patterns of NRMA and NRDA with fixed theta (**c**) and phi (**d**) at λ = 570 nm.

For better visualization, and to mimic experimental observations, we translate this 3D far-field emission pattern into 2D BFP images. This is performed by projecting every *θ* component of the far-field radiation in object space (spherical coordinates) into a *ρ* component (cylindrical coordinates) in the BFP [14,41], as depicted in Figure 2a.

To quantify the directionality of the antennas from the obtained BFP images, we calculate the *F*/*B* ratio. Different definitions can be used to calculate it (see description in the SI and comparison in Figure S1a). Here, we use the following:

(1) $$F/B=10\log_{10}\frac{\int_{\theta_{1}-\delta_{1}}^{\theta_{1}+\delta_{1}}\int_{\varphi_{1}-\delta_{2}}^{\varphi_{1}+\delta_{2}}S(\theta ,\;\varphi )\sin\theta \;d\theta d\varphi }{\int_{\theta_{2}-\delta_{1}}^{\theta_{2}+\delta_{1}}\int_{\varphi_{2}-\delta_{2}}^{\varphi_{2}+\delta_{2}}S(\theta ,\;\varphi )\sin\theta \;d\theta d\varphi }(\mathrm{dB}),$$

where *S* (*θ*, *φ*) represents the power radiated by the antenna in a given direction (*θ*, *φ*) per unit solid angle. Considering the distribution of the signal, we calculate the ratio of radiated power in two broad angular ranges ((*θ*_1_ − *δ*_1_→*θ*_1_ + *δ*_1_, *φ*_1_ − *δ*_2_→*φ*_1_ + *δ*_2_) and (*θ*_2_ − *δ*_1_→*θ*_2_ + *δ*_1_, *φ*_2_ − *δ*_2_→*φ*_2_ + *δ*_2_)) to quantify the *F*/*B* ratio from Equation (1). Here, (*θ*_1_, *φ*_1_) corresponds to the angular position of the maximum lobe in the range 90° < *φ* < 270°, whereas (*θ*_2_, *φ*_2_) is the direction of maximum signal in *φ* ≥ 270° or *φ* ≤ 90°. If there is no lobe in that second angular region, then *φ*_2_ = *φ*_1_ + π. Considering the angular extent of the signal in the simulated BFP images, we chose *δ*_1_ = 10°, *δ*_2_ = 50°. The area enclosed within these values, used for the calculation of the antennas’ *F*/*B* ratio, is marked with red sectors in Figure 2a. Using this definition, we calculate the *F*/*B* ratio as a function of wavelength for the NRDAs. Moreover, since directivity is a key factor in the description of antennas in radio wave applications [42,43], we also take this parameter into account (see comparison with *F*/*B* values in Figure S1a):

(2) $$Dir_{\max}=\frac{4\pi S_{\max}(\theta ,\;\varphi )}{\int_{0}^{2\pi }\int_{0}^{\pi }S(\theta ,\;\varphi )\sin\theta d\theta d\varphi },$$

where *Dir*_max_ represents the ratio of maximum radiated power per unit solid angle *S*_max_ (*θ*, *φ*) to the average radiated power in a 4π direction.

A comparison of these two parameters (*F*/*B* and *Dir*_max_) as a function of wavelength between NRMAs and NRDAs is shown in Figure 2b. For the case of the NRMA, the directivity is around *Dir*_max_ ≈ 7 (or *Dir*_max_ ≈ 1.5 in the absence of a substrate, see Figure S2c, as expected for an infinitesimal dipole antenna [42]) and the *F*/*B* ratio is nearly 0 dB, showing no preferential emission direction. Conversely, for the NRDA, both *Dir*_max_ and *F*/*B* ratio show a peak at λ = 570 nm. To gain insight into the origin of the spectral peak in both magnitudes, we calculate the NRMA and NRDA scattering spectra under plane wave excitation (see Figure S2a). Since the transverse mode of the AuNRs is weaker than the longitudinal one, and both are spectrally close due to the small aspect ratio of AuNRs (1.7), only one scattering peak is observed. Due to mode hybridization [44,45,46], the longitudinal plasmon in the NRMA splits into two bands in the dimer spectrum: One at a short wavelength, the so-called antibonding mode, that emerges at higher energy than the monomer peak and is bright, as the fields along both AuNRs are in in-phase. Another one at a longer wavelength, the bonding mode, that is lower in energy and dark, as the fields along the AuNRs are in antiphase [47,48]. The latter does not show up in the scattering spectra due to the side-to-side symmetry of the dimer when excited by a plane wave. However, in the case of the asymmetrical near-field excitation produced by a nanoemitter placed at the tip of one AuNR, this constraint is removed, and the antiphase mode becomes apparent in the radiated power spectrum, as shown in Figure S3. Similarly to Pakizeh and Käll [27], we observe the maximum directivity near this antiphase mode. A peak in the *F*/*B* ratio appears at λ = 570 nm, which is redshifted with respect to the longitudinal mode of the NRMA, and therefore can then be attributed to the dimer antiphase mode. We also calculate the radiation efficiency, defined as the ratio between radiated power and accepted power, of both NRMA and NRDA (for a near-field dipole excitation at the AuNR tip), and found that latter presents a lower radiation efficiency (see Figure S2b). We associate this effect to the dark character of the antiphase mode that governs the response of the NRDA under this particular excitation. The spectral dependence of both the *F*/*B* ratio and the radiation efficiency, explain why in the experimental conditions [38] the bandpass filter and fluorescence band of the nanoemitter are matched with the antiphase mode of the NRDA. Otherwise, the maximum directionality would be reduced (see Figure S4).

Another way to visualize the radiation pattern of the ultracompact antennas is to use polar plots. Figure 2c shows the azimuthal polar plot (*φ* = 0 to 360°) in the direction of maximum emission (*θ* = 138°) for both NRMAs and NRDAs. Conversely, Figure 2d displays the altitudinal polar plot (*θ* = 0 to 180°) at the direction of maximum emission (*φ* = 356° for NRMAs and 189° for NRDAs). Due to the near-field interaction between the glass surface and the localized surface plasmon of the AuNRs, most of the evanescent field is radiated into the direction corresponding to the critical angle [49,50,51,52] of air–glass interface, *θ_c_* ≈ 42°.

In order to optimize directionality under realistic experimental conditions, we tune several parameters for the dimer antenna. As it is well known, it is hard to controllably orient the dipole moment of emitters, such as fluorescent dyes [53,54,55]. Thus, we first study the effect of the emitter orientation, described as a point dipole. Models of NRDAs with three possible orthogonal dipole orientations are shown in Figure S5a, together with their corresponding BFP images at λ = 570 nm (antiphase mode). By computing the radiation efficiency for each case, we observe that the emitter is not quenched only when it is oriented along the axial direction of the AuNRs (“Orientation 1”, see Figure S5b). For the two other orientations, the radiation power accounts for less than 1% of the power averaged over dipole orientations. Therefore, the average *F*/*B* ratio detectable on the far-field is determined by “Orientation 1”, see Figure S5c.

**Figure 3:**
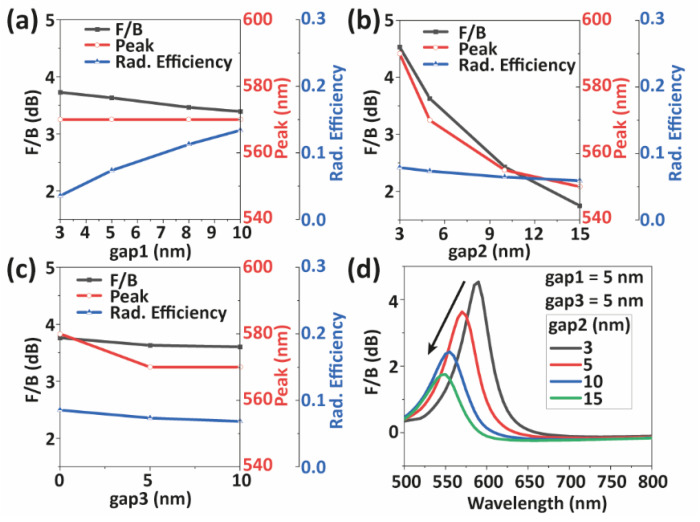
Impact of the gap1, gap2 and gap3 on the *F/B* ratio, peak position and radiation efficiency of NRDAs. (**a**) Variation of gap1 (3, 5, 8, 10 nm). (**b**) Variation of gap2 (3, 5, 10, 15 nm). (**c**) Variation of gap3 (0, 5, 10 nm). (**d**) Spectral dependency of the *F*/*B* ratio for variation of gap2.

Unlike NRMAs, NRDAs are more prone to exhibit deviations from the designed geometry under realistic fabrication conditions. This has an effect on the localized surface plasmon resonances they support too [46]. Therefore, we study the influence of the different geometrical parameters in their optical properties: gap1 plays an important role in the interaction between the AuNRs and the nanoemitter; gap2 controls the extent of the hybridization between the resonant fields sustained by both AuNRs; and gap3 determines the coupling between the antenna and the substrate. According to Figure 3, variations of gap1 (see Figure 3a) and gap3 (see Figure 3c) in the ~10 nm range barely affect the NRDA directionality, showing the robustness of the design with respect to these two parameters. Conversely, reducing gap2 causes a stronger hybridization between the two AuNRs, which gives rise to a redshift of the antiphase NRDA mode. This yields a significant improvement in the *F*/*B* ratio, as shown in Figure 3b,d. Directionality is reduced but not fully lost even for the largest gap considered. We also notice that decreasing gap1 causes a significant non-radiative loss due to higher energy transfer and ohmic dissipation in the AuNRs, an effect that is not affected by changes in gap2 and gap3.

**Figure 4:**
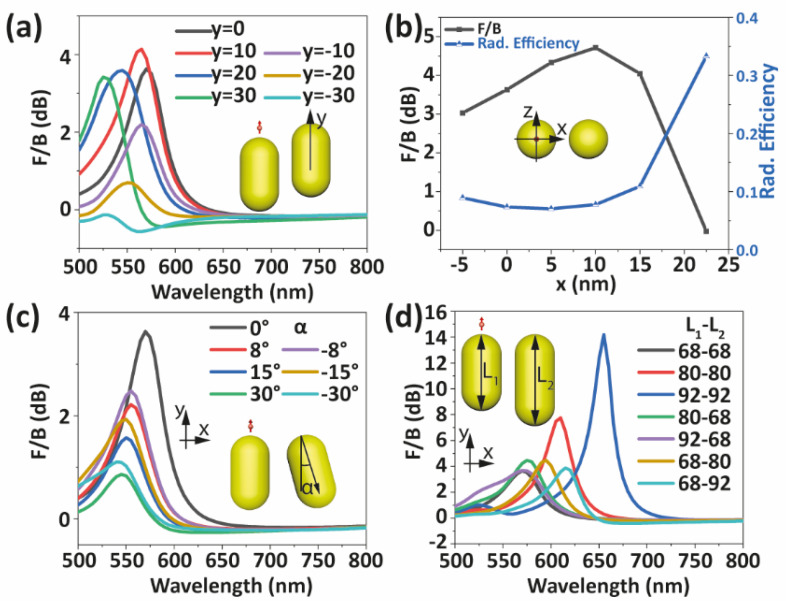
Impact of different configurations on the *F*/*B* ratio of NRDAs. (**a**) The second AuNR moving along y direction. (**b**) *F/B* ratio and radiation efficiency at λ = 570 nm when the nanoemitter is moved along the x direction. Origin point (x = 0) is at the tip center of the first AuNR and x = 22.5 nm corresponds to the top center of the gap between the AuNRs. (**c**) Rotation of one of the AuNRs. Positive (negative) *α* indicates that the second AuNR is rotated anticlockwise (clockwise). (**d**) Variation of the AuNR lengths. The two numbers in the legend represent the length of the first and the second AuNR, respectively, in nanometers.

Other geometrical effects whose experimental control, through synthesis or fabrication, are challenging are: translocation of one AuNR (see Figure 4a), out of plane (x-z) movement of the nanoemitter away from the tip center (see Figure 4b and Figure S7b), rotation of one AuNR (see Figure 4c) and size mismatch between both AuNRs (see Figure 4d). Figure 4 shows that, despite these geometrical changes, most non-optimal dimers still display emission directionality. The *F*/*B* ratio varies only in 0.5 dB when the second AuNR is displaced along the y-direction, from 0 to 30 nm, see Figure 4a. On the contrary, when this second AuNR is moved down along the y-direction, the *F*/*B* ratio goes to 0 dB, and emission direction is even reversed, whereas the radiation efficiency of the antenna increases (see Figure S7a). On the other hand, the *F*/*B* ratio increases when the nanoemitter is displaced towards the second AuNR along the x-direction, presents a maximum and then decreases, vanishing at the top center of the gap between the AuNRs due to symmetry constraints (see Figure 4b). This result is different from those reported for two stacked nanodisks [27], which showed that directionality increased when the nanoemitter is located far away from the gap region between the nanodisks. We attribute this difference to the dissimilar inhomogeneous charge density distribution in the nanoparticles, with their particular shape in each case. Note that the distribution of the induced charges in the AuNRs is barely affected when the nanoemitter is moved along the z-direction (see Figure S7b).

According to Figure 4c, tilting one of the AuNRs has a noticeable effect on both the magnitude of the directionality as well as on the wavelength of the antiphase mode, effects that are always detrimental for the maximum *F*/*B* ratio attainable. Enlarging the length of the NRMA leads to a redshift of the longitudinal mode, and so it does for the antiphase mode of the dimer [46]. Moreover, Figure 4d shows that the *F*/*B* ratio gets significantly increased in such a case. The radiation efficiency at the wavelength of maximum directionality did not change significantly (see Figure S7d), which guarantees detection in experimental conditions. Interestingly, the radiation efficiency spectrum shows a dip at lower wavelengths that is related to enhanced coupling strength and to a larger energy split between the in-phase and antiphase modes in the dimer. Besides, if only one of the AuNRs becomes longer, the maximum *F*/*B* ratio changes only slightly, as shown in Figure 4d.

**Figure 5:**
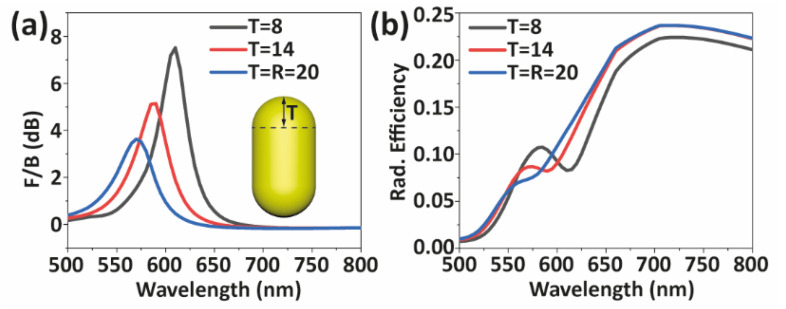
Effect of the AuNR tip curvature on directionality (**a**) and radiated efficiency (**b**) of NRDA. Insets indicate the cap of the AuNR. The total length of the AuNR is kept constant, compressing hemisphere to semi-ellipsoid to control the length of protruding tip (T). Units of T are in nanometers.

Finally, the last geometrical parameter that we analyze is the curvature of the AuNR tips, which produces different local electric fields and severely influences interaction with the nearby nanoemitter [56]. We simulate the curvature of an AuNR by adding semi-spherical caps that have a radius of T = 20 nm. Then, we modify the tip curvature by changing the length of the protrusion (T) and compressing the caps into a semi-ellipsoid shape while keeping the AuNR total length constant (68 nm). We find that not only the longitudinal antiphase mode shifts from 570 nm to 610 nm, but also directionality changes from 3.6 dB to 7.5 dB, as shown in Figure 5a. Moreover, the radiation efficiency at the wavelength of maximum *F*/*B* increases by 13% (see Figure 5b).

## 4. Discussion

**Figure 6:**
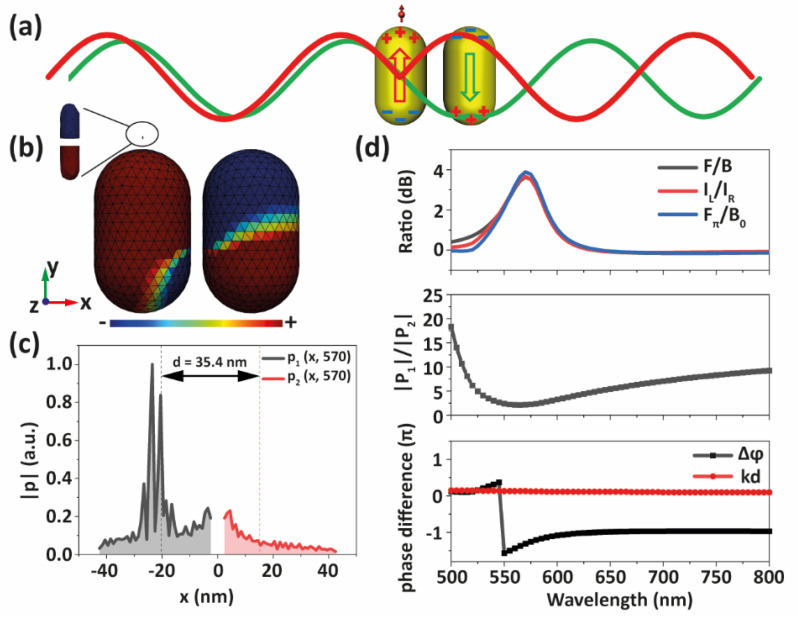
Two-dipole model. (**a**) Schematic representation of the directional emission. Red and green lines represent the electric fields produced by the AuNR close to the dipole nanoemitter and by the second AuNR, respectively. Constructive and destructive interference happen on left and right sides, respectively. (**b**) Surface charge density distribution of model at λ = 570 nm (inset shows the dipole nanoemitter). (**c**) Dipole moment distributions of AuNRs along x direction at λ = 570 nm (see Methods). Black and red dashed lines correspond to average center of dipole moment in the first (left AuNR) and the second (right AuNR) dipole. (**d**) *F*/*B* calculated by: simulation (black line), two-dipole model (red line) and simulated intensity ratio (blue line) at *φ* = π and *φ* = 0 (top), ratio of the magnitude of total dipole moment in both AuNRs (middle) and phase difference (bottom).

Thanks to the strong plasmon hybridization between both AuNRs, the phase delay taking place at the nanometric gap of the NRDA is large enough to replace the larger gaps necessary in Yagi–Uda antennas (required to achieve far-field constructive and destructive interference effects), as illustrated in Figure 6a. In order to explore the mechanism behind this phenomenon in further detail, we utilize a two-dipole analytical model [20] to quantify this phase difference between AuNRs. Once energy has been transferred from the nanoemitter to the NRDA in the near-field, photons are emitted through localized surface plasmons, which are collective oscillations of conduction electrons in the AuNRs. Here, we treat these localized resonances as radiating electric point dipoles. Due to the asymmetric position of the nanoemitter in the NRDA, each AuNR sustains a different electric dipole moment. Thus, the overall system can be described by a dipole moment ratio (*|P*_1_*|*/*|P*_2_*|*), a phase delay originating from plasmon hybridization (∆*φ*) and a phase delay at the gap (*kd*, *k* = 2π*n*/λ, with *n* being the refractive index of the surrounding medium). The gap here is the distance between the location of the AuNR dipole moments, and not gap2, the physical distance between the surfaces of both AuNRs. With these parameters, we asses directionality through the ratio between the intensity of the dipole fields at both sides (left and right) of the dimer:

(3) $$\frac{I_{L}}{I_{R}}=10\log_{10}\frac{{\left|P_{1}+P_{2}\exp(+ikd)\right|}^{2}}{{\left|P_{1}+P_{2}\exp(-ikd)\right|}^{2}}(\mathrm{dB}),$$

According to the simulated surface charge density distribution at the NRDA antiphase mode (λ = 570 nm) shown in Figure 6b, the dipole moments induced at the resonant fields at the two AuNRs present different phases and amplitudes, in clear contrast with the symmetric excitation by a plane wave, see Figure S8a. Induced charge distributions at other wavelengths are also shown in Figure S8. In order to evaluate phase delay *kd* and ∆*φ*, we calculate the dipole moment (*p*) as a function of the position x along the NRDA from the surface charge density distribution in the two AuNRs (see Methods for details). For the NRDA, the gap between the center of both dipole moment distributions is around 35.4 nm, shorter than the distance between the geometric center of the AuNRs (45 nm). This can be linked to inhomogeneous charge distribution on the AuNRs surface, as shown in Figure 6c. The phase in the second AuNR starts to reverse at a wavelength (λ = 550 nm) that corresponds to the hybridized antiphase mode of the dimer. Considering the contributions of the total phase difference and dipole moment ratio, the optimal directionality is at λ = 570 nm, where (*kd − ∆φ*) = 1.43π and *|P*_1_*|*/*|P*_2_*|* = 2.16, see Figure 6d. These *I_L_*/*I_R_* results, obtained from our two-dipole model, are in agreement with FEM simulations, *F*/*B* and *F_π_*/*B_0,_* the latter corresponding to simulated radiated power ratio at *φ* = π and *φ* = 0. Therefore, we can conclude that the directionality of the dimer antenna stems from the antiphase mode under asymmetric near-field excitation by the nanoemitter.

As in the case of the FEM simulations, using the two-dipole model we can analyze the influence of the different geometrical parameters on the behavior of the system. For longer AuNRs (L = 92 nm, see Figure S9), the ratio between both dipole moments (*|P*_1_*|*/*|P*_2_*|*) is closer to 1 (1.06) and (*kd* − *∆φ*) = 1.23π. Hence, the antenna shows a much higher directionality at the resonant wavelength, in agreement with FEM simulations: the *F/B* ratio goes from 3.6 dB (L = 68 nm) to 14.2 dB (L = 92 nm). To some extent, hybridization in a dimer of longer AuNRs is stronger and induces more pronounced interference effects in NRDA emission. As shown in Figures S10-S13, changes in other parameters, such as the tip curvature, the glass substrate or the surrounding medium also show good agreement between FEM simulations and the analytical two-dipole model. Our results indicate that the proposed ultracompact NRDA shows a higher *F*/*B* ratio when the ratio between both dipole moments (*|P*_1_*|*/*|P*_2_*|*) is closer to 1 and (*kd* − ∆*φ*) is closer to π at the antiphase mode, which requires a strong mode hybridization across the gap of the dimer.

## 5. Conclusions

In summary, we have investigated theoretically the experimental feasibility of ultracompact antennas based on two parallel AuNRs, a design that has been recently realized experimentally [38]. We have shown that this design displays robust and excellent directionality within tolerable deviation from the target configuration. Furthermore, the stronger the hybridization between both AuNRs is, the higher the directionality of the antenna, which requires a sufficiently small gap of nanorod dimer. The most crucial aspect of this ultracompact antenna geometry is the precise placement of the single nanoemitter in the near-field of one of the AuNRs, which is indispensable for asymmetric excitation of the anti-phase mode. Our results show that, in contrast to the original design by Pakizeh and Käll [27], the maximum *F/B* ratio is achieved when the nanoemitter is not positioned on top of one of the nanorods but rather shifted towards the center of the antennas. This level of nanometer positioning control can be achieved, for example, via soft template assembly techniques such as DNA origami [57,58], as recently demonstrated [38]. Overall, the ultracompact nanorod dimer design analyzed here provides some guidelines in optimizing directionality of antennas and provides a new possibility to further study antenna-assisted directional single-photon sources for integrated photonic chips.

## Data Availability

Data underlying the results presented in this paper are not publicly available at this time but may be obtained from the authors upon reasonable request.

## Supplementary Materials

The following supporting information can be downloaded at: https://www.mdpi.com/article/10.3390/nano12162841/s1, Figure S1: Methods for calculating the *F*/*B* ratio; Figure S2: Optical properties of NRMAs and NRDAs in air or on air–glass interface; Figure S3: Enhanced radiated power of antenna (NRMA or NRDA) compared with free dipole; Figure S4: Back focal plane images with bandpass filter; Figure S5: Effect of dipole orientation on the NRDA emission; Figure S6: Effect of the local dielectric environment on the *F*/*B* ratio and on the radiation efficiency of NRDA; Figure S7: NRDAs with different configurations impact radiation efficiency; Figure S8: Surface charge density distribution of NRDA under plane wave excitation and excited by a nearby nanoemitter; Figure S9: Effect of longer AuNRs on the two-dipole model results; Figure S10: Effect of absence of a glass substrate on two-dipole model results; Figure S11: Effect of the curvature of the AuNRs on the two-dipole model results; Figure S12: Effect of soaking in water on the two-dipole model results; Figure S13: Effect of smaller gap2 on the two-dipole model results.

## References

[1] Koenderink A.F. (2017). Single-Photon Nanoantennas. ACS Photonics.

[2] Kullock R., Ochs M., Grimm P., Emmerling M., Hecht B. (2020). Electrically-driven Yagi-Uda antennas for light. Nat. Commun..

[3] Filter R., Slowik K., Straubel J., Lederer F., Rockstuhl C. (2014). Nanoantennas for ultrabright single photon sources. Opt. Lett..

[4] Singh A., de Roque P.M., Calbris G., Hugall J.T., van Hulst N.F. (2018). Nanoscale Mapping and Control of Antenna-Coupling Strength for Bright Single Photon Sources. Nano Lett..

[5] Lee K.G., Chen X.W., Eghlidi H., Kukura P., Lettow R., Renn A., Sandoghdar V., Götzinger S. (2011). A planar dielectric antenna for directional single-photon emission and near-unity collection efficiency. Nat. Photonics.

[6] Novotny L., van Hulst N. (2011). Antennas for light. Nat. Photonics.

[7] Sakat E., Wojszvzyk L., Greffet J.-J., Hugonin J.-P., Sauvan C. (2020). Enhancing Light Absorption in a Nanovolume with a Nanoantenna: Theory and Figure of Merit. ACS Photonics.

[8] Andersen S.K.H., Kumar S., Bozhevolnyi S.I. (2017). Ultrabright Linearly Polarized Photon Generation from a Nitrogen Vacancy Center in a Nanocube Dimer Antenna. Nano Lett..

[9] Baiyasi R., Goldwyn H.J., McCarthy L.A., West C.A., Jebeli S.A.H., Masiello D.J., Link S., Landes C.F. (2021). Coupled-Dipole Modeling and Experimental Characterization of Geometry-Dependent Trochoidal Dichroism in Nanorod Trimers. ACS Photonics.

[10] Chen W., Roelli P., Hu H., Verlekar S., Amirtharaj S.P., Barreda A.I., Kippenberg T.J., Kovylina M., Verhagen E., Martinez A. (2021). Continuous-wave frequency upconversion with a molecular optomechanical nanocavity. Science.

[11] Xomalis A., Zheng X., Chikkaraddy R., Koczor-Benda Z., Miele E., Rosta E., Vandenbosch G.A.E., Martinez A., Baumberg J.J. (2021). Detecting mid-infrared light by molecular frequency upconversion in dual-wavelength nanoantennas. Science.

[12] Saemisch L., Liebel M., van Hulst N.F. (2020). Control of Vibronic Transition Rates by Resonant Single-Molecule-Nanoantenna Coupling. Nano Lett..

[13] Tanaka Y.Y., Kimura T., Shimura T. (2021). Unidirectional emission of phase-controlled second harmonic generation from a plasmonic nanoantenna. Nanophotonics.

[14] Curto A.G., Volpe G., Taminiau T.H., Kreuzer M.P., Quidant R., van Hulst N.F. (2010). Unidirectional Emission of a Quantum Dot Coupled to a Nanoantenna. Science.

[15] Kosako T., Kadoya Y., Hofmann H.F. (2010). Directional control of light by a nano-optical Yagi–Uda antenna. Nat. Photonics.

[16] See K.M., Lin F.C., Chen T.Y., Huang Y.X., Huang C.H., Yesilyurt A.T.M., Huang J.S. (2018). Photoluminescence-Driven Broadband Transmitting Directional Optical Nanoantennas. Nano Lett..

[17] Abedi S., Pakizeh T. (2017). Packed Yagi-Uda nano-antennas using a unidirectional feed at visible wavelengths. Opt. Lett..

[18] Shegai T., Johansson P., Langhammer C., Kall M. (2012). Directional scattering and hydrogen sensing by bimetallic Pd-Au nanoantennas. Nano Lett..

[19] Shegai T., Chen S., Miljkovic V.D., Zengin G., Johansson P., Kall M. (2011). A bimetallic nanoantenna for directional colour routing. Nat. Commun..

[20] Vercruysse D., Sonnefraud Y., Verellen N., Fuchs F.B., Di Martino G., Lagae L., Moshchalkov V.V., Maier S.A., Van Dorpe P. (2013). Unidirectional Side Scattering of Light by a Single-Element Nanoantenna. Nano Lett..

[21] Vercruysse D., Zheng X., Sonnefraud Y., Verellen N., Di Martino G., Lagae L., Vandenbosch G.A., Moshchalkov V.V., Maier S.A., Van Dorpe P. (2014). Directional fluorescence emission by individual V-antennas explained by mode expansion. ACS Nano.

[22] Lu G., Wang Y., Chou R.Y., Shen H., He Y., Cheng Y., Gong Q. (2015). Directional side scattering of light by a single plasmonic trimer. Laser Photonics Rev..

[23] Lai Y.H., Cui X.M., Li N.N., Shao L., Zhang W., Wang J.F., Lin H.Q. (2021). Asymmetric Light Scattering on Heterodimers Made of Au Nanorods Vertically Standing on Au Nanodisks. Adv. Opt. Mater..

[24] Pezeshki H., Wright A.J., Larkins E.C. (2021). Ultra-compact and ultra-broadband hybrid plasmonic-photonic vertical coupler with high coupling efficiency, directivity, and polarisation extinction ratio. IET Optoelectron..

[25] Taminiau T.H., Stefani F.D., van Hulst N.F. (2008). Enhanced directional excitation and emission of single emitters by a nano-optical Yagi-Uda antenna. Opt. Express.

[26] Kasani S., Curtin K., Wu N. (2019). A review of 2D and 3D plasmonic nanostructure array patterns: Fabrication, light management and sensing applications. Nanophotonics.

[27] Pakizeh T., Käll M. (2009). Unidirectional Ultracompact Optical Nanoantennas. Nano Lett..

[28] Liu M., Lee T.-W., Gray S.K., Guyot-Sionnest P., Pelton M. (2009). Excitation of Dark Plasmons in Metal Nanoparticles by a Localized Emitter. Phys. Rev. Lett..

[29] Shen H., Lu G., He Y., Cheng Y., Gong Q. (2017). Unidirectional enhanced spontaneous emission with metallo-dielectric optical antenna. Opt. Commun..

[30] Shen H., Chou R.Y., Hui Y.Y., He Y., Cheng Y., Chang H.-C., Tong L., Gong Q., Lu G. (2016). Directional fluorescence emission from a compact plasmonic-diamond hybrid nanostructure. Laser Photonics Rev..

[31] Bonod N., Devilez A., Rolly B., Bidault S., Stout B. (2010). Ultracompact and unidirectional metallic antennas. Phys. Rev. B.

[32] Zhang T., Xu J., Deng Z.L., Hu D., Qin F., Li X. (2019). Unidirectional Enhanced Dipolar Emission with an Individual Dielectric Nanoantenna. Nanomaterials.

[33] Barreda A.I., Saiz J.M., González F., Moreno F., Albella P. (2019). Recent advances in high refractive index dielectric nanoantennas: Basics and applications. AIP Adv..

[34] Bidault S., Mivelle M., Bonod N. (2019). Dielectric nanoantennas to manipulate solid-state light emission. J. Appl. Phys..

[35] Alaee R., Albooyeh M., Tretyakov S., Rockstuhl C. (2016). Phase-change material-based nanoantennas with tunable radiation patterns. Opt. Lett..

[36] Yao K., Liu Y. (2016). Controlling Electric and Magnetic Resonances for Ultracompact Nanoantennas with Tunable Directionality. ACS Photonics.

[37] Kerker M., Wang D.S., Giles C.L. (1983). Electromagnetic scattering by magnetic spheres. J. Opt. Soc. Am..

[38] Zhu F., Sanz-Paz M., Fernández-Domínguez A.I., Zhuo X., Liz-Marzán L.M., Stefani F.D., Pilo-Pais M., Acuna G.P. (2022). DNA-Templated Ultracompact Optical Antennas for Unidirectional Single-Molecule Emission. Nano Lett..

[39] Johnson P.B., Christy R.W. (1972). Optical Constants of the Noble Metals. Phys. Rev. B.

[40] Huang Y., Ringe E., Hou M., Ma L., Zhang Z. (2015). Near-field mapping of three-dimensional surface charge poles for hybridized plasmon modes. AIP Adv..

[41] Lieb M.A., Zavislan J.M., Novotny L. (2004). Single-molecule orientations determined by direct emission pattern imaging. J. Opt. Soc. Am. B.

[42] Balanis C.A. (2015). Antenna Theory: Analysis and Design.

[43] Bharadwaj P., Deutsch B., Novotny L. (2009). Optical Antennas. Adv. Opt. Photonics.

[44] Prodan E., Radloff C., Halas N.J., Nordlander P. (2003). A hybridization model for the plasmon response of complex nanostructures. Science.

[45] Jain P.K., Eustis S., El-Sayed M.A. (2006). Plasmon Coupling in Nanorod Assemblies:  Optical Absorption, Discrete Dipole Approximation Simulation, and Exciton-Coupling Model. J. Phys. Chem. B.

[46] Basyooni M.A., Ahmed A.M., Shaban M. (2018). Plasmonic hybridization between two metallic nanorods. Optik.

[47] Li J.N., Liu T.Z., Zheng H.R., Gao F., Dong J., Zhang Z.L., Zhang Z.Y. (2013). Plasmon resonances and strong electric field enhancements in side-by-side tangent nanospheroid homodimers. Opt. Express.

[48] Flauraud V., Bernasconi G.D., Butet J., Alexander D.T.L., Martin O.J.F., Brugger J. (2017). Mode Coupling in Plasmonic Heterodimers Probed with Electron Energy Loss Spectroscopy. ACS Nano.

[49] Lukosz W., Kunz R.E. (1977). Light emission by magnetic and electric dipoles close to a plane interface. I. Total radiated power. J. Opt. Soc. Am..

[50] Lukosz W., Kunz R.E. (1977). Light emission by magnetic and electric dipoles close to a plane dielectric interface. II. Radiation patterns of perpendicular oriented dipoles. J. Opt. Soc. Am..

[51] Lukosz W. (1979). Light emission by magnetic and electric dipoles close to a plane dielectric interface. III. Radiation patterns of dipoles with arbitrary orientation. J. Opt. Soc. Am..

[52] Hellen E.H., Axelrod D. (1987). Fluorescence Emission at Dielectric and Metal-Film Interfaces. J. Opt. Soc. Am. B.

[53] Hubner K., Joshi H., Aksimentiev A., Stefani F.D., Tinnefeld P., Acuna G.P. (2021). Determining the In-Plane Orientation and Binding Mode of Single Fluorescent Dyes in DNA Origami Structures. ACS Nano.

[54] Mathur D., Kim Y.C., Díaz S.A., Cunningham P.D., Rolczynski B.S., Ancona M.G., Medintz I.L., Melinger J.S. (2020). Can a DNA Origami Structure Constrain the Position and Orientation of an Attached Dye Molecule?. J. Phys. Chem. C.

[55] Adamczyk A.K., Huijben T.A., Sison M., di Luca A., Chiarelli G., Vanni S., Brasselet S., Mortensen K., Stefani F.D., Pilo-Pais M. (2022). DNA self-assembly of single molecules with deterministic position and orientation. arXiv.

[56] Kern A.M., Martin O.J. (2011). Excitation and reemission of molecules near realistic plasmonic nanostructures. Nano Lett..

[57] Rothemund P.W. (2006). Folding DNA to create nanoscale shapes and patterns. Nature.

[58] Kuzyk A., Jungmann R., Acuna G.P., Liu N. (2018). DNA Origami Route for Nanophotonics. ACS Photonics.

