# Supplementary material for: Optical Ultracompact Directional Antennas Based on a Dimer Nanorod Structure
Source: Nanomaterials (Basel). 2022 Aug 18;12(16):2841. doi: 10.3390/nano12162841 (PMC9416387; doi:10.3390/nano12162841)
Supplement: Supplementary file 1 [file nanomaterials-12-02841-s001.zip › nanomaterials-1857652-supplementary.pdf]

Optical Ultracompact Directional Antennas Based on a Dimer Nanorod Structure

Fangjia Zhu [1,*], María Sanz-Paz [1], Antonio I. Fernández-Domínguez [2], Mauricio Pilo-Pais [1] and Guillermo P. Acuna [1,*]

[1] Department of Physics, University of Fribourg, Chemin du Musée 3, CH-1700 Fribourg, Switzerland
[2] Departamento de Física Teórica de la Materia Condensada and Condensed Matter Physics Center (IFIMAC), Universidad Autónoma de Madrid, E-28049 Madrid, Spain
* Correspondence: fangjia.zhu@unifr.ch (F.Z.); guillermo.acuna@unifr.ch (G.P.A.)

S1. Methods for calculating the *F*/*B* ratio

To quantify the directionality of the antennas from the obtained BFP images, we calculate the *F*/*B* ratio using different definitions:

$$F/B = 10\log_{10}\frac{\int_{\theta_1-\delta_1}^{\theta_1+\delta_1}\int_{\varphi_1-\delta_2}^{\varphi_1+\delta_2} S(\theta,\ \varphi)\sin\theta\ d\theta d\varphi}{\int_{\theta_2-\delta_1}^{\theta_2+\delta_1}\int_{\varphi_2-\delta_2}^{\varphi_2+\delta_2} S(\theta,\ \varphi)\sin\theta\ d\theta d\varphi}\ \ (dB), \tag{S1}$$

$$F_\pi/B_0 = 10\log_{10}\frac{S(\theta_1,\ \pi)}{S(\theta_1,\ 0)}\ \ (dB), \tag{S2}$$

$$F_p/B_p = 10\log_{10}\frac{S(\theta_1,\ \varphi_1)}{S(\theta_1,\ \varphi_1-\pi)}\ \ (dB), \tag{S3}$$

$$F_1/B_2 = 10\log_{10}\frac{S(\theta_1,\ \varphi_1)}{S(\theta_1,\ \varphi_2)}\ \ (dB), \tag{S4}$$

where $S(\theta, \varphi)$ represents the power radiated by the antenna in a given direction (θ, φ) per unit solid angle. Considering the distribution of the signal, we calculate the ratio of radiated power in two broad angular ranges $((\theta_1 - \delta_1 \rightarrow \theta_1 + \delta_1, \varphi_1 - \delta_2 \rightarrow \varphi_1 + \delta_2)$ and $(\theta_2 - \delta_1 \rightarrow \theta_2 + \delta_1, \varphi_2 - \delta_2 \rightarrow \varphi_2 + \delta_2))$ to quantify *F*/*B* from Equation (S1). Here, (θ_1, φ_1) corresponds to the angular position of the maximum lobe in the range $90° < \varphi < 270°$, whereas (θ_2, φ_2) is the direction of maximum signal in $\varphi \geq 270°$ or $\varphi \leq 90°$. If there is no lobe in that second angular region, then $\varphi_2 = \varphi_1 + \pi$. Considering the angular extent of the signal in the simulated BFP images, we chose $\delta_1 = 10°$, $\delta_2 = 50°$.

Similarly, Equation (S2) describes the radiated power ratio between the direction with $\varphi_1 = \pi$ and inverse the direction with $\varphi_2 = 0$, while Equation (S3) computes the radiated power ratio between the direction with maximum radiated power and the inverse direction with same θ. Finally, Equation (S4) outputs the ratio between the radiated power in the direction of maximum lobe in two half spaces.

We calculate the *F*/*B* ratio as a function of wavelength for antennas using the above-described equations. All the obtained values are put together in Figure S1a. It is worth noticing that, regardless of the method used for the *F*/*B* quantification, the maximum value always occurs at the same wavelength (λ = 570nm).

Furthermore, the maximum point in the two lobes are not always in opposite directions, as shown in Figure S1b. For some cases, one point might not be enough to represent the whole intensity on one side. For those reasons, we mainly use *F*/*B* ratio in Equation (S1) to quantify directionality of antenna.

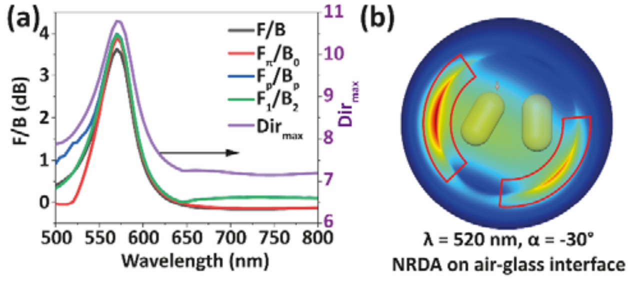

Figure S1. Methods for calculating the *F*/*B* ratio. **(a)** Comparison of different quantification methods. **(b)** Back focal plane image of the NRDA with the first AuNR rotated clockwise 30°.

S2. Optical properties of NRMAs and NRDAs

We calculate the scattering spectra of both NRMA and NRDA under plane wave excitation in air or on an air-glass interface, the antennas' radiation efficiency and Dir_{max} under near-field excitation by a nanoemitter, as shown in Figure S2. The presence of a glass substrate only causes a slight red-shift for both NRMAs and NRDAs.

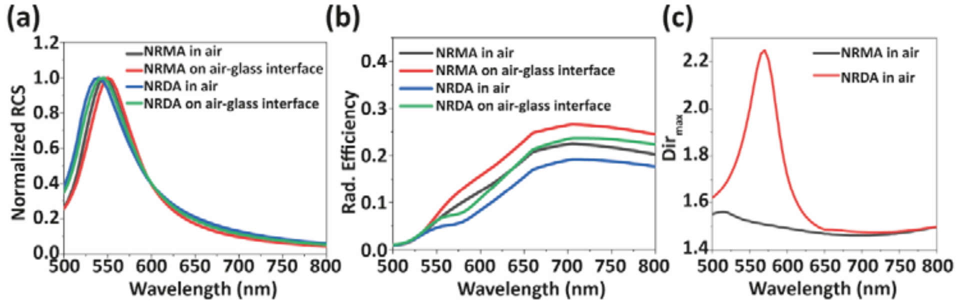

Figure S2. Optical properties of NRMAs and NRDAs in air or on an air-glass interface. **(a)** Normalized scattering spectra of NRMA and NRDA under plane wave excitation. **(b)** Radiation efficiency of NRMA and NRDA under near-field excitation. **(c)** Maximum directivity of NRMA and NRDA in air under near-field excitation.

S3. Radiated power enhancement in the presence of the antenna

Apart from the *F*/*B* ratios, we also compare the radiated power of a nanoemitter in the presence (P_r) and absence (P_{r0}) of the antennas, as shown in Figure S3.

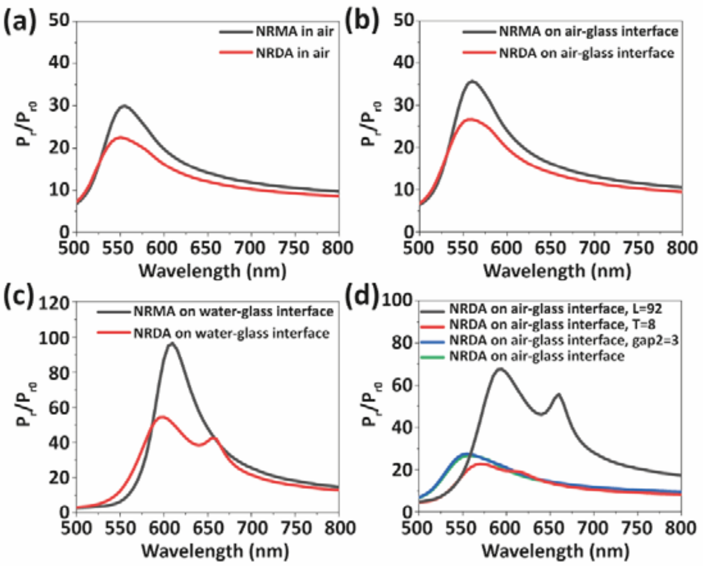

Figure S3. Enhanced radiated power of antenna (NRMA or NRDA) compared to free dipole. Enhancement effect of NRDA and NRMA in air **(a)** and on an air-glass interface **(b)**. **(c)** Enhancement effect of NRDA and NRMA on a water-glass interface. **(d)** Enhancement effect of NRDA with different size, flattened AuNR and gap2.

S4. Spectral dependence of antennas' directionality

Taking the spectral differences in radiation efficiency of the antenna into account, we calculate the *F*/*B* ratio within some bandpass filters (Figure S4). If no bandpass filter is used, the final directionality gets reduced by nearly half: *F*/*B* ratio goes from 3.3 dB in the case of a bandpass filter located at the antiphase mode, to 1.5 dB when collecting all the spectral signal (540 -710 nm).

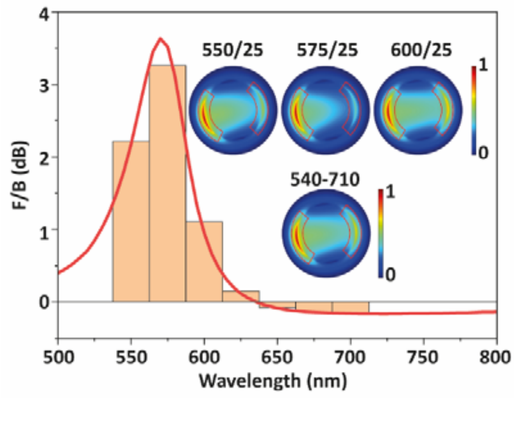

Figure S4. Back focal plane images with bandpass filters. Red line represents the *F*/*B* ratio of NRDA at every wavelength and the histogram represents the *F*/*B* ratio within a 25nm bandpass filter. Inserts are back focal plane images of 550/25, 575/25, 600/25 and broad spectral ranges.

S5. Effect of dipole orientation on the NRDA emission

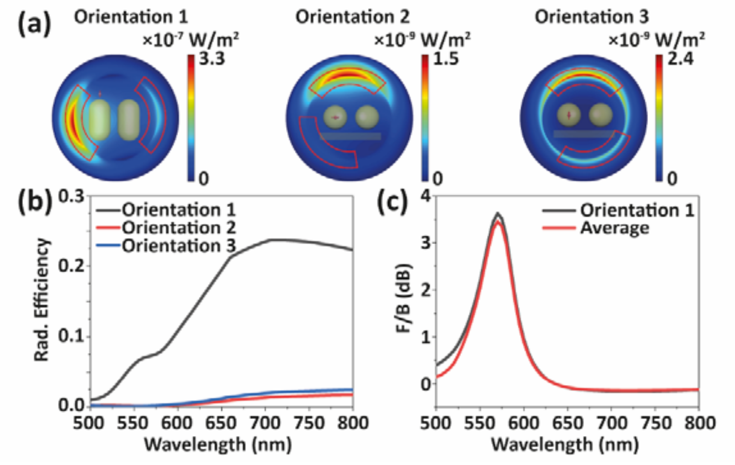

Figure S5. Effect of dipole orientation on the NRDA's emission. (a) Schematics of the dipole orientation and corresponding back focal plane images at λ = 570 nm. (b) Radiation efficiency of NRDAs for different dipole orientations. (c) Spectral dependency of the F/B ratio for a dipole with "Orientation 1" and for an averaged rotated dipole.

S6. Effect of the local dielectric environment on the *F*/*B* ratio and on the radiation efficiency of NRDA

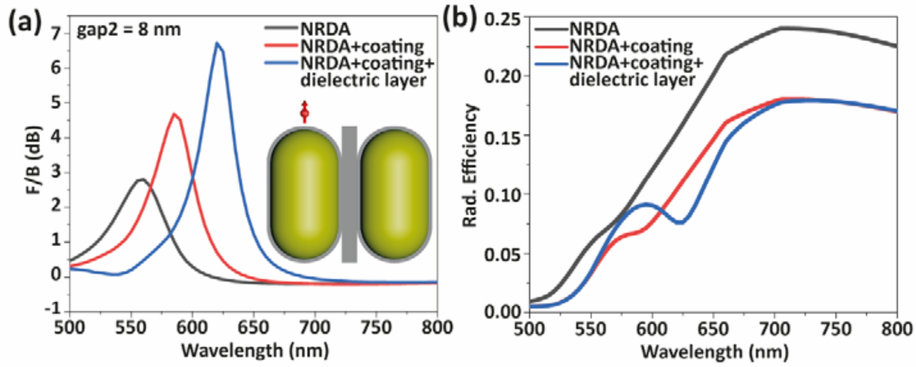

Figure S6 Effect of the local dielectric environment on the *F*/*B* ratio (**a**) and on the radiation efficiency (**b**) of NRDAs. Gap2 is set to 8 nm, the thickness of the coating layer (DNA) is 2 nm and its refractive index is set to 1.7. The size of the dielectric layer (representing DNA origami) is 72 × 40 × 4 nm (length × width × thickness) and its refractive index is set to 2.1.

S7. Radiation efficiency of NRDAs as a function of the configuration

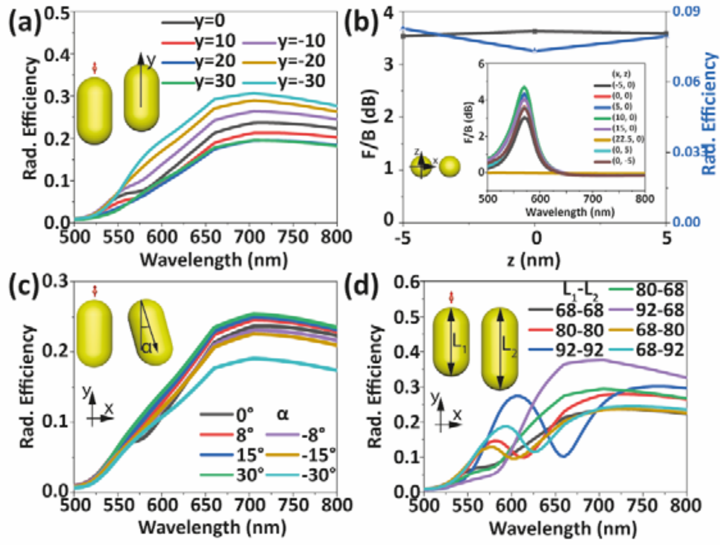

Figure S7. NRDAs with different configurations' impacts on radiation efficiency. **(a)The** second AuNR moves along y direction. **(b)** F/B and radiation efficiency at $\lambda = 570$ nm when the nanoemitter moves on the top of the AuNR along z direction. Inset shows the F/B curve when the nanoemitter moves along x and z direction. **(c)** Positive α represents an anticlockwise rotation of the first AuNR around its vertex in plane. Negative α represents a clockwise rotation of the first AuNR around its vertex in plane. **(d)** Combination of AuNRs with different lengths. The two numbers represent the length of the first and the second AuNR, in order. Units of T are in nanometers.

S8. Surface charge density distribution of NRDA under plane wave excitation and excited by a nearby nanoemitter

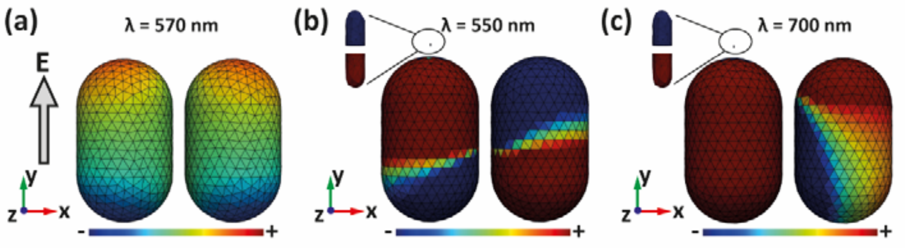

Figure S8. Surface charge density distribution of NRDA under plane wave excitation and excited by a nearby nanoemitter. **(a)** Surface charge density distribution of NRDA excited by 570 nm plane wave in air. Surface charge density distribution of NRDA at **(b)** $\lambda = 550$ nm, **(c)** $\lambda = 700$ nm on an air-glass interface excited by a nearby emitter.

S9. Effect of AuNRs' length, glass substrate, tip curvature and surrounding medium on the two-dipole model results

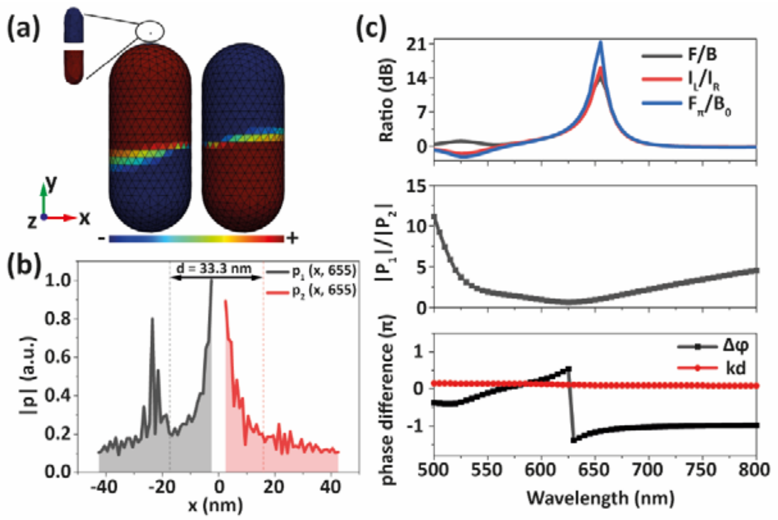

Figure S9. Effect of longer AuNRs on the two-dipole model results. **(a)** Surface charge density distribution of the model at λ = 655 nm (on an air-glass interface, L = 92 nm). **(b)** Dipole moment distributions of AuNRs along x direction at λ = 655 nm. Black and red dash lines correspond to the average center of the dipole moment in the first (left AuNR) and the second (right AuNR) dipoles. **(c)** *F*/*B* ratios calculated by: simulation (black line), two-dipole model (red line) and simulated intensity ratio (blue line) at $\varphi = \pi$ and $\varphi = 0$ (top), ratio of the magnitude of total dipole moment in both AuNRs (middle) and phase difference (bottom).

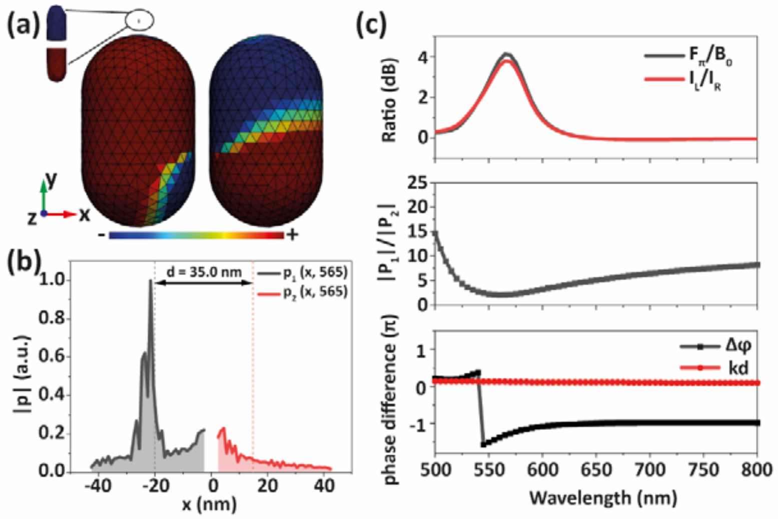

Figure S10. Effect of the absence of a glass substrate on the two-dipole model results. **(a)** Surface charge density distribution of the model at λ = 565 nm (in air). **(b)** Dipole moment distributions of the AuNRs along x direction at λ = 565 nm. Black and red dash lines correspond to the average center of the dipole moment in the first (left AuNR) and the second (right AuNR) dipoles. **(c)** *F*/*B* ratios calculated by: simulated intensity ratio (black line) at $\varphi = \pi$ and $\varphi = 0$ (black line) and two-dipole model (red line) (top), ratio of the magnitude of total dipole moment in both AuNRs (middle) and phase difference (bottom).

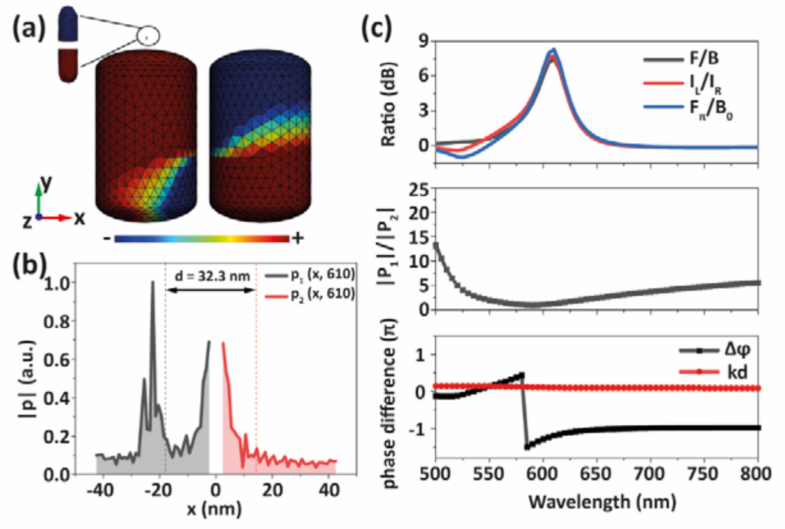

Figure S11. Effect of the curvature of the AuNRs on the two-dipole model results. **(a)** Surface charge density distribution of the model at λ = 610 nm (on air-glass interface, T = 8 nm). **(b)** Dipole moment distributions of the AuNRs along x direction at λ = 610 nm. Black and red dash lines correspond to average center of dipole moment in the first (left AuNR) and the second (right AuNR) dipoles. **(c)** *F*/*B* ratios calculated by: simulation (black line), two-dipole model (red line) and simulated intensity ratio (blue line) at $\varphi = \pi$ and φ = 0 (top), ratio of the magnitude of total dipole moment in both AuNRs (middle) and phase difference (bottom).

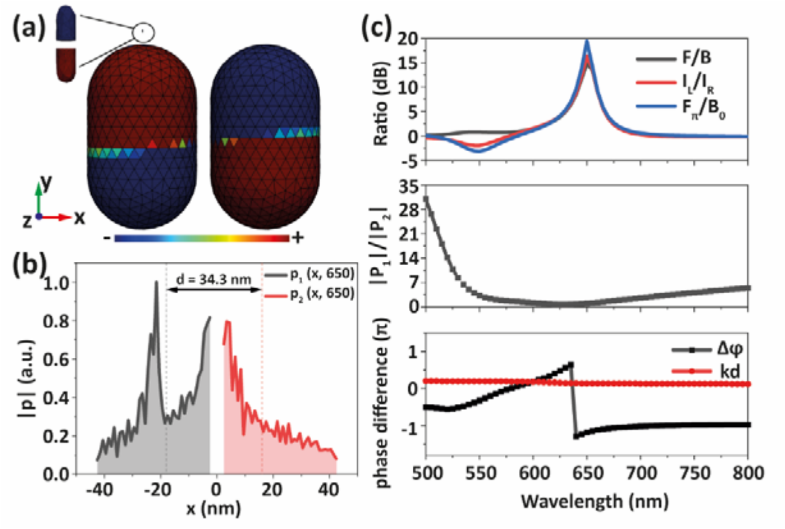

Figure S12. Effect of soaking in water on the two-dipole model results. **(a)** Surface charge density distribution of the model at λ = 650 nm (on water-glass interface). **(b)** Dipole moment distributions of the AuNRs along x direction at λ = 650 nm. Black and red dash lines correspond to average center of dipole moment in the first (left AuNR) and the second (right AuNR) dipoles. **(c)** *F*/*B* ratios calculated by: simulation (black line), two-dipole model (red line) and simulated intensity ratio (blue line) at $\varphi = \pi$ and φ = 0 (top), ratio of the magnitude of total dipole moment in both AuNRs (middle) and phase difference (bottom).

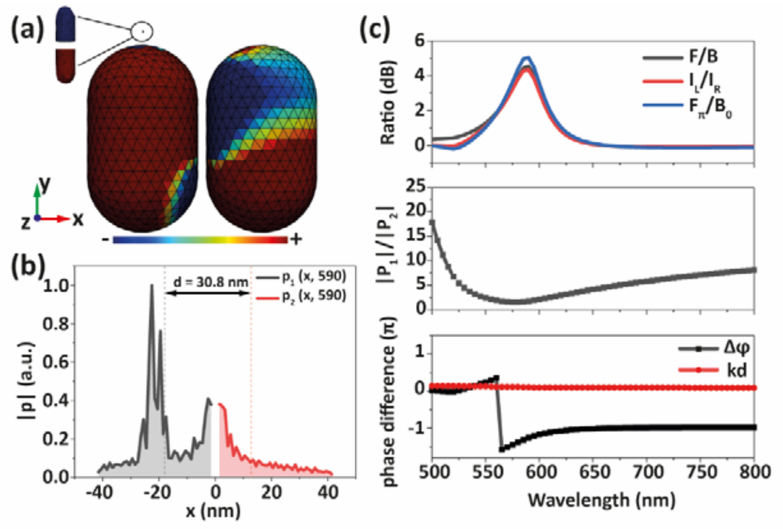

Figure S13. Effect of smaller gap2 on the two-dipole model results. **(a)** Surface charge density distribution of the model at λ = 590 nm (on air-glass interface, gap2 = 3 nm). **(b)** Dipole moment distributions of the AuNRs along x direction at λ = 590 nm. Black and red dash lines correspond to average center of dipole moment in the first (left AuNR) and the second (right AuNR) dipoles. **(c)** *F*/*B* ratios calculated by: simulation (black line), two-dipole model (red line) and simulated intensity ratio (blue line) at $\varphi = \pi$ and $\varphi = 0$ (top), ratio of the magnitude of total dipole moment in both AuNRs (middle) and phase difference (bottom).
